# Asymmetric Preparation of *α*-Quaternary Fluorinated *β*-keto Esters. Review

**DOI:** 10.3390/molecules25143264

**Published:** 2020-07-17

**Authors:** Albert Granados, Adelina Vallribera

**Affiliations:** Department of Chemistry and Centro de Innovación en Química Avanzada (ORFEO-CINQA), Carrer dels Til.lers, UAB Campus, Universitat Autònoma de Barcelona, 08193 Barcelona, Spain

**Keywords:** fluorination, trifluoromethylation, trifluoromethylthiolation, *β*-keto esters, asymmetric

## Abstract

In this review, recent advances over the past decade in the preparation of fluorinated stereogenic quaternary centers on *β*-keto esters compounds are analyzed. Since the incorporation of fluorine and fluorinated groups is of special interest in pharmaceutical chemistry, a range of metal-catalyzed and organocatalyzed methods have been developed. Herein, we review the enantioselective fluorination, trifluoromethylation and trifluoromethylthiolation of 3-oxo esters. The scope, the induction of enantioselectivity and mechanistic investigations are presented.

## 1. Introduction

The field of organofluorine chemistry has been of increasing interest to academia and industry during recent decades [1,2,3]. Fluorine now occupies a prestigious position, especially in the design of biologically active compounds, and indeed, nearly 30% of human medicines and 35% of agrochemicals on the market contain one or more fluorine atoms [4,5,6]. The incorporation of fluorine atoms into the drug discovery process has emerged as a common strategy [7]. The physicochemical effects imparted by fluorine explain its utility and impact in a wide range of drugs, including antidepressant, antipsychotic, antitumor, antiviral, anaesthetic, anti-inflammatory agents, etc. [8,9,10]. The inclusion of a fluorine atom in a drug molecule can have a profound effect on the molecule’s pharmacodynamic, pharmacokinetic, distribution and extent of drug metabolism [11,12]. For example, the addition of functional groups to therapeutic molecules can greatly enhance the lipophilicity of the drug, which in turn can enhance bioavailability, tissue distribution and cell permeability. Also, it can affect the interaction of the drug with the pharmacological target for example, through effects on the inter and intramolecular forces present in the binding [13,14].

In addition, the use of radioactive ^18^F nucleus in positron emission topography (PET) labelling broadened the applicability in medicine, serving as a privileged diagnostic tool for cancer and other diseases [15]. The use of [^18^F]fluorodeoxyglucose (FDG) and other [^18^F]labelled radiotracers in oncology, brain diseases and cardiology has established the value of ^18^F as a positron emitter for PET and encouraged new developments for late stage fluorination. Furthermore, to enable early monitoring of various diseases with non-invasive diagnostic tools ^19^F magnetic resonance imaging (MRI) has been exploited with great success. Applications can be found in targeted imaging of selected relevant physiological techniques, in targeted drug delivery and cell tracking. ^19^F MRI agents have been classified in molecular tracers, polymers and branched derivatives [16].

Fluorinated materials have found applications in materials sciences and their importance in modern industry and technology is expected to grow even more in the near future [17]. Fluorinated materials have been widely used in liquid crystals [18,19], smartphone displays, photovoltaic solar cells, surface modification chemistry [20], stabilization of metal nanoparticles [21,22] and crystal/metal-organic frameworks (MOFs) engineering. Introduction of fluorous tags onto materials can favour ordering, self-organization, hydrophobicity [23] and chemical stability. Fluorinated polymers exhibit outstanding chemical resistance, thermal stability, low friction coefficients and electrical properties [24]. In particular some fluorinated polymers have been intensively investigated and applied due to their electroactive properties in biomedical applications including controlled drug delivery systems and tissue engineering.

Thus, due to its importance, the development of new methodologies that give access to fluorinated compounds is in demand [25,26,27]. In particular, the stereoselective introduction of fluorine or fluoroalkylated groups to generate stereogenic centres has been of growing interest. Specifically, as we will see throughout this manuscript, the selective fluorination at specified positions of an organic compound forming C-F, C-CF_3_ and C-SCF_3_ bonds from C-H is especially important and a challenging task. 

There are plenty of papers published in the past two decades in reference to the asymmetric introduction of these groups into organic molecules and therefore is impossible to include all of them. However, there are excellent reviews that should not be omitted [28,29,30,31]. Since 2000, a practical enantioselective methodology leading to construction of a C−F quaternary stereocenter has been developed using chiral ***N***−F reagents derived from *N*-fluoroammonium salts by the combination of cinchona alkaloids and Selectfluor^®^. A great diversity of silyl enol ethers, allylsilanes, 1,3-dicarbonyl compounds, oxindoles, dipeptide, and enolates can be used as substrates. Other methods include primary and secondary amine catalysts via enamine intermediates, cationic and anionic phase-transfer catalyst, etc. [28]. At the same time, in 2010 an important breakthrough in this chemistry related to a metal catalytic protocol was the pioneering research by using Ti/TADDOL catalyst with Selectfluor^®^ [28,32]. These protocols are based on electrophilic fluorinating processes. The comprehensive discussion of the catalytic asymmetric scenario for C-F formation, including organocatalytic methods and transition-metal catalyzed transformations, has been previously reviewed [28,29,30]. Moreover, the development of approaches for the straightforward asymmetric introduction of trifluoromethyl groups into small molecules, including nucleophilic, electrophilic, or free radical processes, has received much recent attention [29,30]. In contrast to their nucleophilic counterparts (generally based on the use of Ruppert–Prakash reagent TMSCF_3_), enantioselective and radical electrophilic trifluoromethylation reactions remain far less developed. In particular, MacMillan and co-workers described a conceptually novel approach to the asymmetric α-trifluoromethylation of aldehydes via the merger of enamine catalysis, CF_3_I and photoredox catalysis [33]. Allen and MacMillan also reported in 2010 the highly asymmetric α-trifluoromethylation of aldehydes using Togni’s reagent utilizing both enamine organocatalysis and transition metal catalysis [34]. For a historical background on the most important useful methods for trifluoromethylthiolation please see an excellent review of Billard [31]. While this group has been utilized for some time, it is only very recently that methods for its direct asymmetric introduction have come into the mainstream and will be revised herein.

In this review, evolution and advances over the last ten years in the preparation of fluorinated quiral quaternary centres on *β*-keto esters compounds are analysed. Simple *β*-keto esters have been selected and studied by many groups and are well established as substrates in synthesis. Possessing an active methylene and two functional groups make them really versatile molecules in organic synthesis [35,36,37]. They are useful synthetic precursors, as for example the ketone is easily converted to other functional groups (i.e., alcohols and imines) and the ester can be transformed to different amides through the carboxylic acid. The utility of the enantioselective fluorinated *β*-keto esters has been highlighted by highly diastereoselective transformations of the trifluoromethylated products carried out by Gade’s group [38]. The scope, the induction of enantioselectivity and mechanistic investigations of C*_α_*-F, C*_α_*-CF_3_ and C*_α_*-SCF_3_ bonds formation in *β*-keto esters are presented.

## 2. Asymmetric Preparation of Quaternary C-F Stereocentres on *β*-keto Esters

**Figure 1:**
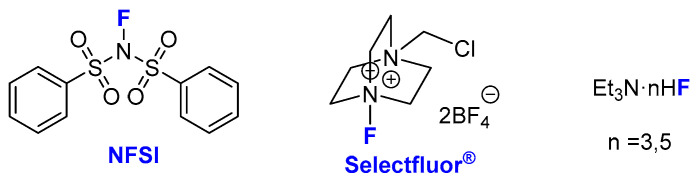
Mostly common electrophilic and nucleophilic fluorinating reagents.

At first glance, to fluorinate the *α*-intercarbonylic position of a *β*-keto ester an electrophilic reagent will be necessary. The development of stable sources of electrophilic fluorinating agents (Figure 1), such as *N*-fluorobenzensulfonimide [39] (NFSI) and Selectfluor^®^ [40] has had a great impact and witnessed encouraging progress on the catalytic enantioselective electrophilic fluorination. On the contrary, an enantioselective nucleophilic fluorination of *β*-keto esters will be an umpolung reaction and is still in its beginnings because of the low reactivity of the fluoride anion. Recently nucleophilic fluoride sources as Et_3_N·3HF and Et_3_N·5HF have been used for the enantioselective oxidative fluorination of *β*-keto esters [41,42].

### 2.1. Metal-Catalyzed Methods

**Figure 2:**
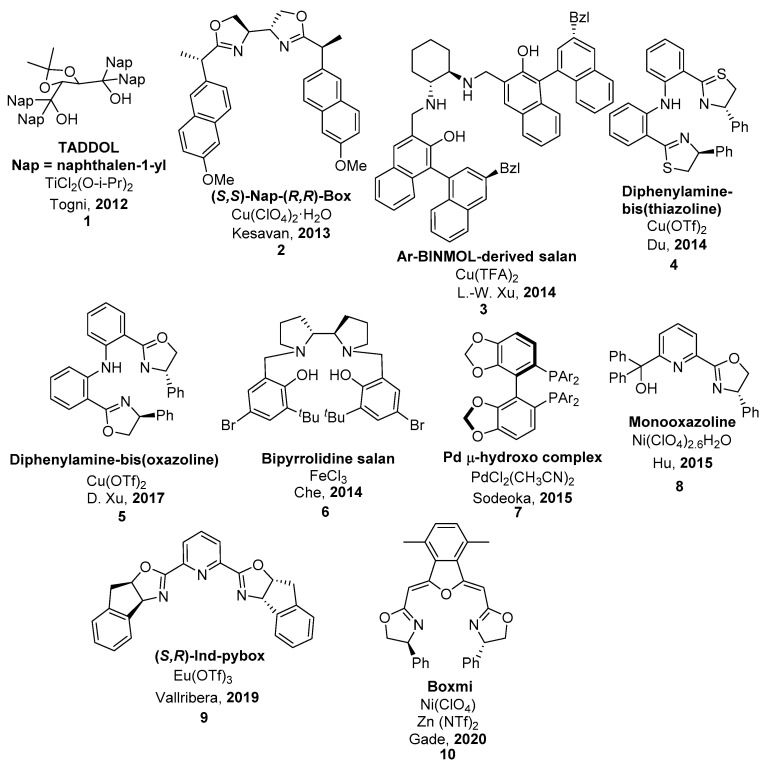
Chiral ligands and metals combinations for the catalytic enantioselective fluorination of *β*-keto esters.

An important breakthrough in this chemistry occurred when Togni and Hintermann described the catalytic *α*-fluorination reactions of α-substituted acyclic *β*-keto esters using a Ti/TADDOL complexes (**1**, Figure 2) and Selectfluor^®^ (Figure 1), yielding the fluorinated compounds in 62–90% *ee* [32]. 

**Scheme 1:**
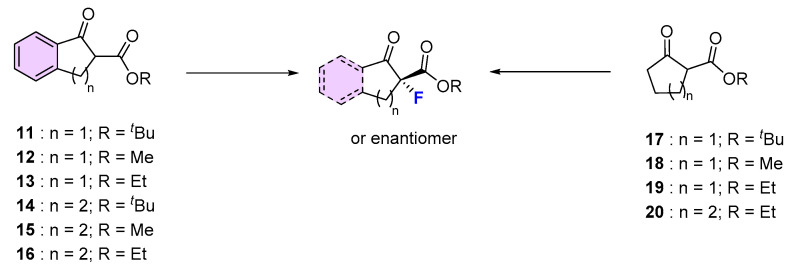
Cyclic *β*-keto esters **11**–**20**.

Although there was only one substrate which gave an excellent 90% *ee*, they demonstrated that chiral metal complexes could promote the stereoselective fluorination. Ti/TADDOL complexes acted as Lewis acid activating the *β*-keto ester. From this report until 2010 a range of metal catalysed fluorination reactions have been developed and previously reviewed [43]. Some years later, in 2012, Togni’s group in a remarkable paper mainly dedicated to acyclic 1,3-dicarbonyl compounds, reported that using the same Ti/TADDOL complex the cyclic tetralone **16** (Scheme 1), gave the *α*-fluorinated product in 20% *ee* (entry 20, Table 1) [44]. We will comment on these results on acyclic 1,3-dicarbonyl later on.

In addition to titanium, copper complexes have been also used as Lewis acids. The group of Kesavan studied the combination of Cu(II)/(*S*,*S*)-Nap-(*R*,*R*)-Box (**2**, Figure 2) with NFSI as a fluorinating reagent, achieving a 34% *ee* in the *α*-fluorination of ethyl 1-indanone-2-carboxylate **13** Upon enlarging the ring size to the cyclohexanone derivative (compound **16**) the *ee* decreased to 16% (entries 11 and 21, Table 1). On the contrary, some alkyl 2-oxocyclopentane-1-carboxylates **17**–**19** (Scheme 1) gave higher enantioselective values (72–86% *ee*) [45]. A notable advantage is that ester bulkiness is not needed to achieve high *ee*. Hexafluoro-2-propanol (HFIP) was used as additive to increase the enantioselectivities, promoting the release of the fluorinated product from the catalyst as others have reported before [46]. 

**Figure 3:**
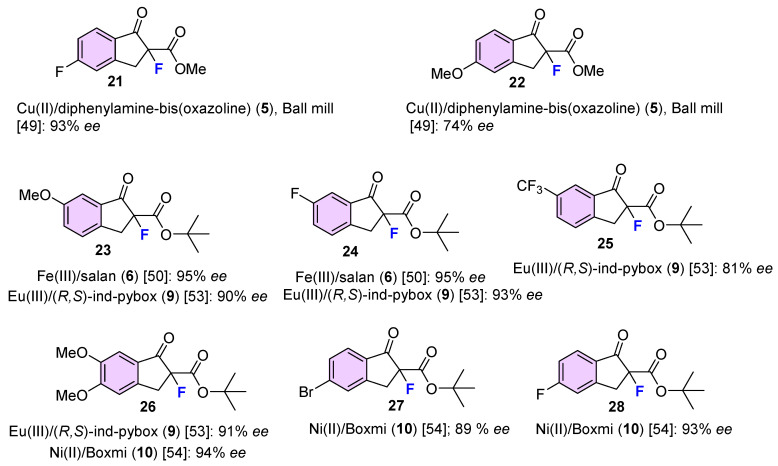
Results for substituted derivatives **21**–**28.**

If we keep talking about copper, asymmetric fluorination of methyl 1-indanone-2-carboxylate (**12**) catalyzed by the Cu(II)/Ar-BINMOL-derived salan system (Ar-BINMOL = 1,1′-binaphthalene-2-*α*-arylmethanol-2′-ol) (**3**, Figure 2) gave 82% *ee* and 99% yield (entry 10, Table 1) [47]. Only *β*-keto ester **12** (Scheme 1) was tested, however, these conditions were applied with great success to a series of *β*-keto amides. In addition to this work, efficient enantioselective fluorination of *β*-keto esters and amides, catalysed by Cu(II)/diphenylamine-linked bis(thiazoline) complexes (**4**, Figure 2), was achieved [48]. These conditions work nicely with five (**11**, **13,** Scheme 1) and six membered rings (**15**, **16,** Scheme 1) and with different esters (entries 5, 15, 18 and 22, Table 1). Unfortunately, racemic product (46% yield) was obtained with **19** as substrate, and compound **20** did not react under these conditions (entries 29 and 32, Table 1). In 2017, Xu and collaborators reported a fast and highly enantioselective fluorination method for different alkyl 1-indanone-2-carboxylates catalysed by a chiral non-commercial diphenylamine Box and Cu(OTf)_2_ (**5**, Figure 2) [49]. The reactions were conducted using a ball mill apparatus (Fritsch Planetary Micro Mill model “Pulverisette 7”) in the absence of solvent, yielding the fluorinated compounds with enantioselectivities up to 99% *ee* (15 examples, 74–99% *ee*). Ester functionalities with different steric hindrance were well tolerated (entries 3, 8 and 17, Table 1). Halogen substitutions on the aromatic ring gave excellent induction, whereas electron-donating substituents dropped the *ee* (**21** and **22**, Figure 3). With a six-member ring substrate (compound **15**) the *ee* dropped to 56% (entry 17 of Table 1). In addition, compounds **19** and **20** (Scheme 1) gave excellent results in terms of reactivity and *ee* under these conditions (entries 30 and 33, Table 1). 

Chiral Fe(III)-salan complexes (**6**, Figure 2) with AgClO_4_ (2 mol.%) as additives and NFSI, catalysed the reaction of *t*-butyl 1-indanone-2-carboxylates with different substituents in the aromatic ring (11 examples, 94–97% *ee*). The bulkiness of the ester group played an important role, as less hindered esters gave lower *ee* (46–79% *ee*) (compare entries 1, 6 and 12, Table 1). The influence of different substituents in the aromatic position does not have much influence on the *ee* (**23** and **24**, Figure 3). With six membered ring substrate (**14**) the *ee* dropped to 69% (entry 16, Table 1). Remarkably, acyclic *t*-butyl keto esters gave excellent *ee* (87–94%), as did *t*-butyl 2-oxocyclopentane-1-carboxylate, **17** (95% *ee*, entry 24, Table 1). Addition of silver salt accelerated the reaction [50]. 

In 2015, Pd μ-hydroxo dimeric complexes containing (*R*)-DTBM-SEGPHOS (Ar = 3,5-(*t*-Bu)_2_-4-MeOC_8_H_2_) (**7**, Figure 2) with NFSI in *i*PrOH gave 90% *ee* in the fluorination of *t*-butyl 2-oxocyclopentane-1-carboxylate (**17**) as substrate (only one example, entry 25, Table 1). The method was extensively applied to *β*-keto amides [51]. In the same year chiral mono-oxazoline ligands ((*S*)-diphenyl(6-(4-phenyl-4,5-dihydrooxazol-2-yl)pyridine-2-yl)methanol) and Ni(ClO_4_)_2_·6H_2_O (**8**, Figure 2) in DCM using NFSI, catalysed the reaction of different alkyl 1-indanone-2-carboxylates (10 examples, 71–78% *ee*). Different methyl and halogens substituents on the aromatic ring were tested, showing low influence on the results. Curiously enough the *t*-butyl ester **11** (Scheme 1) gave a racemic fluorination reaction (entry 2 of Table 1), as did methyl 2-oxocyclopentane-1-carboxylate (**18**, entry 26 of Table 1). Moreover, ethyl 1-oxo-1,2,3,4-tetrahydronaphtalene-2-carboxylate (**15**) gave a low 13% *ee* (entry 19 of Table 1) [52]. 

**Table 1:** Conditions and results for enantioselective fluorination reactions of compounds of Scheme 1 (from 2010 to 2020).

Entry	Substrate	Fluorinating Reagent	Pre-Catalyst (see Figure 2)	Yield (%)	*ee* (%)	Ref
1	**11**	NFSI	Fe(III)/salan (**6**) 0 °C, MeCN, AgClO_4_ (2 mol.%)	96	94	[50]
2	**11**	NFSI	Ni(II)-monooxazoline (**8**)	89	0	[52]
3	**11**	NFSI	Cu(II)/diphenylamine-bis(oxazoline) (**5**), Ball mill	99	95	[49]
4	**11**	NFSI	Eu(III)/(*R*,*S*)-ind-pybox (**9**)	78	96 (*S*)	[53]
5	**11**	NFSI	Cu(II)/diphenylamine-linked bis(thiazoline) (**4**), CHCl_3_, rt	93	**99** (*S*)	[48]
6	**12**	NFSI	Fe(III)/salan (**6**)0 °C, MeCN, AgClO_4_ (2 mol.%)	99	46	[50]
7	**12**	NFSI	Ni(II)-monooxazoline (**8**)	90	75	[52]
8	**12**	NFSI	Cu(II)/diphenylamine-bis(oxazoline), (**5**), Ball mill	97	92	[49]
9	**12**	NFSI	La(III)/(*R*,*S*)-ind-pybox (**9**)	80	62 (*S*)	[53]
10	**12**	NFSI	Cu/Ar-BINMOL-derived salan (**3**) Xylene, 0 °C	99	**82** (*S*)	[47]
11	**13**	NFSI	Cu(II)/(*S*,*S*)-Nap-(*R*,*R*)-Box (**2**) 0 °C, toluene, HFIP	98	34	[45]
12	**13**	NFSI	Fe(III)/salan (**6**) 0 °C, MeCN, AgClO_4_ (2 mol.%)	99	59	[50]
13	**13**	NFSI	Ni(II)-monooxazoline (**8**)	85	77	[52]
14	**13**	NFSI	Cu(II)/diphenylamine-bis(oxazoline) (**5**), Ball mill	99	91	[49]
15	**13**	NFSI	Cu(II)/diphenylamine-linked bis(thiazoline) (**4**), CHCl_3_, rt	100	**99**	[48]
16	**14**	NFSI	Fe(III)/salan (**6**) 0 °C, MeCN, AgClO_4_ (2 mol.%)	96	**69**	[50]
17	**15**	NFSI	Cu(II)/diphenylamine-bis(oxazoline) (**5**), Ball mill	93	56	[49]
18	**15**	NFSI	Cu(II)/diphenylamine-linked bis(thiazoline) (**4**), CHCl_3_, rt	100	**93**	[48]
19	**15**	NFSI	Ni(II)-monooxazoline (**8**)	86	13	[52]
20	**16**	Selectfluor^®^	Ti/TADDOL (**1**), rt, MeCN	93	20	[44]
21	**16**	NFSI	Cu(II)/(*S*,*S*)-Nap-(*R*,*R*)-Box (**2**) 0 °C, toluene, HFIP	90	16	[45]
22	**16**	NFSI	Cu(II)/diphenylamine-linked bis(thiazoline) (**4**), CHCl_3_, rt	99	**93**	[48]
23	**17**	NFSI	Cu(II)/(*S*,*S*)-Nap-(*R*,*R*)-Box (**2**) 0 °C, toluene, HFIP	97	83	[45]
24	**17**	NFSI	Fe(III)/salan (**6**) O °C, MeCN, AgClO_4_ (2 mol.%)	88	**95**	[50]
25	**17**	NFSI	Pd(II)/(*R*)-DTBM-SEGPHOS (7) *i*PrOH, rt	93	90	[51]
26	**18**	NFSI	Ni(II)-monooxazoline (**8**)	30	0	[52]
27	**18**	NFSI	Cu(II)/(*S*,*S*)-Nap-(*R*,*R*)-Box (**2**) 0 °C, toluene, HFIP	96	**72**	[45]
28	**19**	NFSI	Cu(II)/(*S*,*S*)-Nap-(*R*,*R*)-Box (**2**) 0 °C, toluene, HFIP	93	86	[45]
29	**19**	NFSI	Cu(II)/diphenylamine-linked bis(thiazoline) (**4**), CHCl_3_, rt	46	0	[48]
30	**19**	NFSI	Cu(II)/diphenylamine-bis(oxazoline) (**8**), Ball mill	96	**92**	[49]
31	**20**	NFSI	Cu(II)/(*S*,*S*)-Nap-(*R*,*R*)-Box (**2**) 0 °C, toluene, HFIP	90	52	[45]
32	**20**	NFSI	Cu(II)/diphenylamine-linked bis(thiazoline) (**4**), CHCl_3_, rt	n. r.	-	[48]
33	**20**	NFSI	Cu(II)/diphenylamine-bis(oxazoline) (**5**), Ball mill	95	**99**	[49]

Based on our previous experience [35,36,37], we have established a catalytic method for the highly enantioselective *α*-fluorination of a series of *t*-butyl 1-indanone-2-carboxylates, using europium (III) triflate and (*S*,*R*)-ind-pybox as pre-catalyst (**9**, Figure 2), and NFSI as electrophilic fluorinating agent in acetonitrile at −30 °C (6 examples, 81–96% *ee*). Results revealed a dependence of the enantiocontrol on the steric hindrance of the ester groups in substrates. In general, comparing substituent’s with similar steric hindrance, the presence of electron withdrawing groups in the benzene harmed the *ee* (**23** and **25**, Figure 3). Access to both enantiomers of the *α*-fluorinated oxo ester is guaranteed by the commercial availability of both (*R*,*S*) and (*S*,*R*) ind-pybox C2-symmetric ligands [53].

Very recently, Gade’s group reported a remarkable contribution using Boxmi ligands in combination with Ni(II) and Zinc (II) complexes (**10**, Figure 2) with NFSI reagent. They screened four *t*-butyl indanone-2-carboxylates substituted in the aromatic ring with Ni(II)/Boxmi obtaining excellent *ee* values (89–94% *ee*). Methyl ester gave worse results (64% *ee*). Electron-rich substituents, such as the methoxy group in the aromatic ring of 1-indanone-2-carboxylate, caused slight increased the *ee* (Figure 3). Three new examples with other substituents in the aromatic ring were tested with Zn(II)/Boxmi and *t*-butyl ester giving also high levels of induction (84–92%) [54]. 

All these different reported methodologies are summarized in Table 1. Indanone **11** possessing a *t*-butyl ester nicely reacted (78–99 yield and 94–99 *ee*) under four different pre-catalysts combination (entries 1,3-5). Lower inductions are obtained with less bulky methyl and ethyl esters (compounds **12** and **13,** Scheme 1). The best general conditions are the ones described by Xu and collaborators which consist in using the Cu(II)/diphenylamine-bis(oxazoline) combination in a ball mill apparatus [49]. We also recommend the Cu(II)/diphenylamine-linked bis(thiazoline) combination [48]. Generally, alkyl 1-oxo-1,2,3,4-tetrahydronaphtalene-2-carboxylates **14**–**16** gave lower *ee* than their indanyl analogues **11**–**13** (Scheme 1) (compare entries 1 and 16, and entries 8 and 17, Table 1), except in the case of Cu(II)/diphenylamine-linked bis(thiazoline) combination [48]. Alkyl 2-oxocyclopentane-1-carboxylates **17** and **18** normally gave the fluorinated products in lower *ee*. However, with Fe(III)/salan an excellent 95% *ee* was reported for substrate **17** having a *t*-butyl ester [50]. From Table 1 the combination of Cu(II)/diphenylamine-bis(oxazoline) in a ball mill system is recommended for **19** and **20** (Scheme 1), affording 92 and 99% *ee*, respectively. In general, for all the methodologies (Figure 3) electron-rich substituents such as the methoxy group in the aromatic ring of 1-indanone-2-carboxylate caused increases in *ee.*

**Figure 4:**
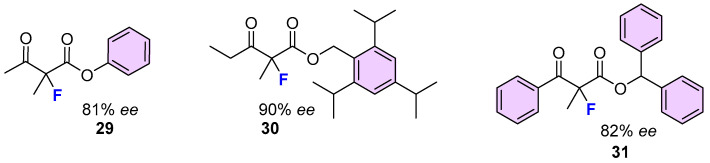
Fluorinated acyclic *β*-keto esters prepared by Togni’s research group [44].

**Figure 5:**
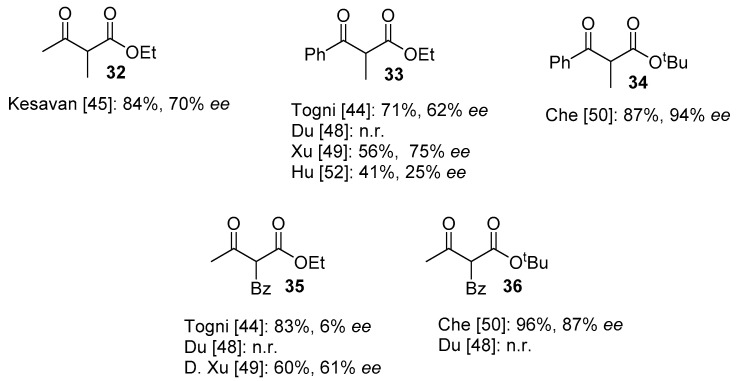
Examples of acyclic *β*-keto esters used for validation of methodologies. Reaction conditions are summarized in Table 1 and Figure 2; all the results correspond to optimized conditions.

Acyclic *β*-keto esters are difficult substrates in this reaction even in terms of reactivity. Regarding the enantioinduction they are not so rigid and have more degrees of conformational freedom in comparison with cyclic 3-oxo esters. In 2012, Togni’s group reported [44] that using the Ti/TADDOL complexes (**1**, Figure 2) and Selectfluor^®^ (Figure 1) a series of alkyl *α*-methyl 3-oxobutanoates were fluorinated giving excellent yields (44–96%) and moderate *ee* (10 examples, 45–81% *ee*). In addition, alkyl *α*-methyl 3-oxopentanoates could be fluorinated with moderate to high selectivities (7 examples, 30–90% *ee*). Alkyl 2-methyl 2-benzoylpropanoate compounds were also examined, giving good results (12 examples, 55–82% *ee*). Best results were obtained with bulky ester groups (**29–31**, Figure 4). Fluorination of ethyl ester **33** gave a moderate 62% *ee* (Figure 5). The scope of this method is the most extensive reported until now. 

Kesavan’s group reported that ethyl 2-methyl-3-oxobutanoate (**32**, Figure 5) underwent fluorination in 84% yield and 70% *ee* (only one example) using the Cu(II)/(*S*,*S*)-Nap-(*R*,*R*)-*Box* (**2**, Figure 2) combination [45]. Du et al. using Cu/diphenylamine-linked bis(thiazoline) (**4**, Figure 2) evaluated the fluorination of some acyclic *α*-alkyl *β*-keto esters, such as ethyl 2-methyl-3-oxo-3-phenylpropanoate (**33**) and ethyl and *t*-butyl 2-benzyl-3-oxobutanoates **35**, **36**. Unfortunately, no fluorinated products were obtained [48]. In addition, Xu’s group reported that using Cu/diphenylamine-linked bis (oxazoline) (**5**, Figure 2) in a ball mill system [49] fluorination of ethyl 2-benzyl-3-oxobutanoate **35** afforded a moderate yield (60%) and *ee* (61%); compound **33** also produced the fluorinated compound in 56% yield and 75% *ee* (Figure 5). Using the Fe(III)-salan combination [50] (**6**, Figure 2), compound **34** reacted nicely, giving 87% yield and 94% *ee*. *β*-Keto ester **36** gave 96% yield and 87% *ee* of the fluorinated derivative (Figure 5). Low yield (41%) and *ee* (25%) was obtained using **33** and the Ni(II)-monooxazoline ligand [52]. 

**Scheme 2:**
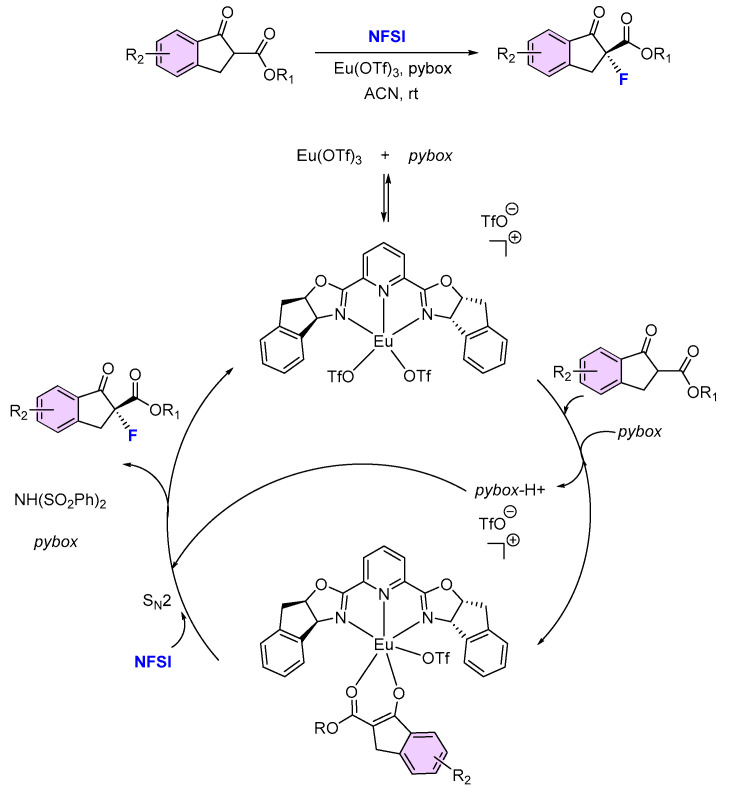
Proposed catalytic cycle [53].

The reactions described above may all proceed through a similar catalytic cycle regardless of the metal complex used. First, coordination to the metal centre increases the acidity of the *α*-proton, allowing the metal enolate to be easily generated. The chiral ligand normally acts as a base. Subsequent reaction affords the formation of a stereogenic C-F bond through a S_N_2 mechanism. The enantioselectivity of the reaction stems from the efficient blockage of one of the faces. See as an example the catalytic cycle proposed for the asymmetric fluorination using europium (III) triflate and (*S*,*R*)-ind-pybox combination [53] (Scheme 2). 

### 2.2. Organocatalytic Methods

**Figure 6:**
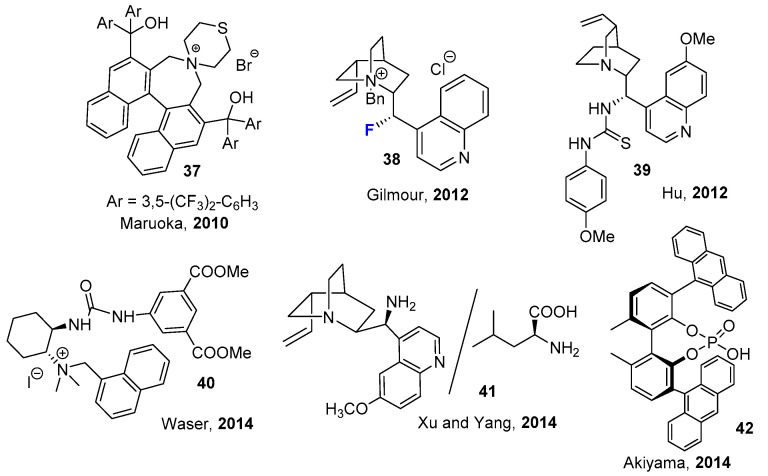
Structure of different organocatalysts.

Phase-transfer catalysis (PTC) has been applied to the enantioselective fluorination of *β*-keto esters. The first example was reported by Kim and Park in 2002 using quaternary ammonium salts from cinchonine (10% mol, Figure 5), NFSI and K_2_CO_3_ [55]. Some years later chiral bifunctional phase transfer catalysts using binaphtyl derivatives **37** (2% mol, Figure 6) were used by Maruoka and collaborators in the fluorination of different *t*-butyl 1-indanone-2-carboxylates affording the products in excellent yields and *ee* (4 examples 94–98% *ee*) using NFSI and K_2_CO_3_ [56]. Substrates **14** and **17** gave 90 and 98% *ee*, respectively. Only bulky *t*-butyl esters were reported [56]. 

In addition, a designing study of fluorinated cinchone alkaloids was carried out by the group of Gilmour [57]. Using optimized PTC **38** (10% mol, Figure 6), *t*-butyl and adamantyl 1-indanone-2-carboxylates gave moderate *ee* (78 and 78% *ee* respectively) with NFSI and Cs_2_CO_3_. Some studies on the use of multifunctional catalyst **41** (Figure 6) combining cinchona alkaloid-derived primary amines (10% mol) with l-leucine (20% mol) gave good reactivity using Selecfluor^®^, although low *ee* in the fluorination of several alkyl 1-indanone-2-carboxylates (four examples, 39–55% *ee*) [58]. 

**Figure 7:**
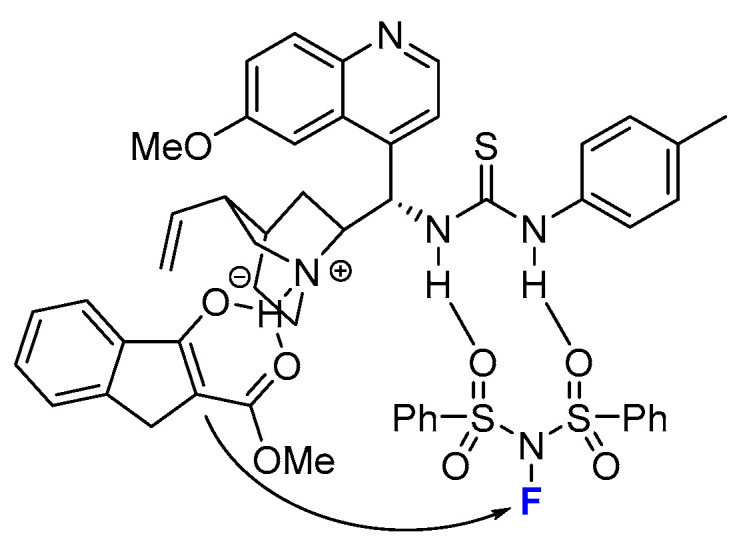
Plausible intermediate proposed by Hu [59].

In 2012, bifunctional chiral thioureas **39** (10% mol, Figure 6) with NFSI, DMAP as a base at −60 °C were used as organocatalysts in the enantioselective fluorination of different alkyl 1-indanone-2-carboxylates (11 examples, up to 99% *ee*) [59]. Methyl, ethyl, isopropyl and benzyl ethers gave excellent results, whereas surprisingly *t*-butyl afforded racemic fluorination. Some alkyl 2-oxocyclo-pentane-1-carboxylates were assayed with success. Benzyl 1-oxo-1,2,3,4-tetrahydronaphtalene-2-carboxylate and several acyclic *β*-keto esters gave low *ee*, except in the case of compound **35** that afforded a nice 81% *ee*. A possible reactive intermediate has been proposed by the authors that illustrates this kind of organocatalysis using thioureas (Figure 7). The authors envisioned that the thiourea group was attached to NFSI via hydrogen bonds; and the *β*-keto ester was coordinated with the tertiary amine through a hydrogen bond of its enol form [59]. In addition, the Waser group used urea-containing chiral ammonium salts **40** (2% mol, Figure 6) with NFSI and K_3_PO_4_ as a base at −10 °C affording the fluorination of a small series of alkyl 1-indanone-2-carboxylates (11 examples, 70–86% *ee*). *β*-Keto esters **14** and **17** gave 74 and 78% *ee*, respectively) [60]. 

Akiyama reported in 2014 [61] an interesting work based on the in situ generation of a chiral sodium phosphate derived from a chiral phosphoric acid [**42**, Figure 6]. Two active species (sodium enolate and sodium phosphate) were generated under Na_2_CO_3_ basic conditions. The corresponding *α*-fluoro *β*-keto esters including methyl, ethyl and benzyl esters were obtained in excellent yields with good to excellent *ee*. With Akiyama’s group conditions, the enantioselective fluorination of indanones **12** and **13** was afforded in a 99% and 87% *ee*, respectively. For the *t*-butyl ester only a 20% *ee* was achieved. The method is not useful for tetralone **16**. 

**Scheme 3:**
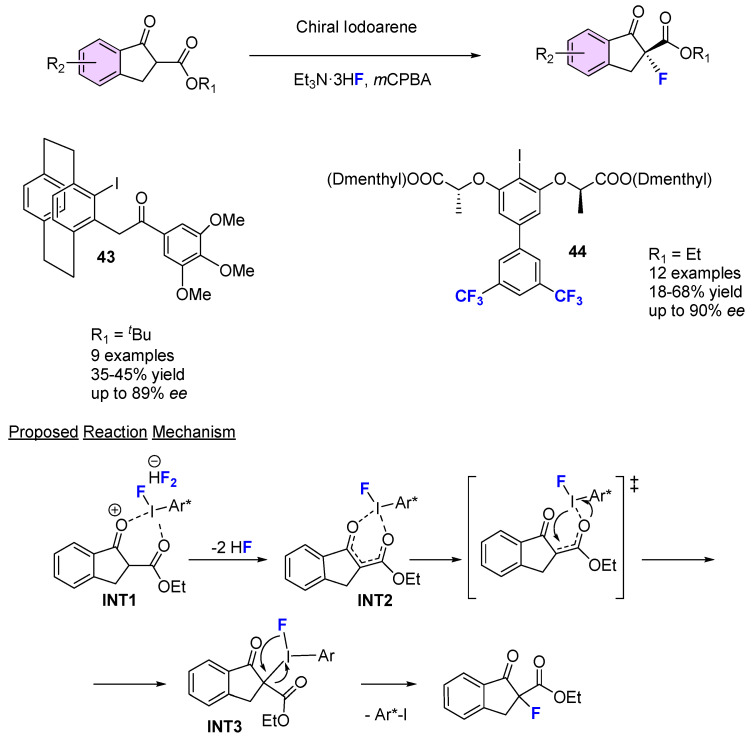
Combination of hydrogen fluoride and an oxidant mediated by an hypervalent iodine compound. Plausible mechanism.

In 2018, Zheng’s group developed a highly enantioselective oxidative method for the fluorination of *β*-keto esters using Et_3_N·3HF as a nucleophilic fluoride source mediated by a chiral iodo arene organocatalyst **43** and *m*CPBA as oxidant. The yields were low in all the studied cases [41]. Some months after Rueping et al. reported a similar strategy using chiral iodoarene **44** obtaining higher yields and enantioselectivities [42] (Scheme 3). A plausible mechanism (Scheme 3) is the in situ formation of Ar*-I-F_2_ by oxidation of the hypervalent iodine reactive with HF and the m-CPBA followed by the coordination with *β*-keto ester (**INT1**). Then, the next step involves the reaction of the enol form of substrate with [Ar-I-F]^+^/[HF_2_]^−^ to abstract the H atom with formation of an O-bonded hypervalent iodine. This underwent a 1,3-migration to afford intermediate **INT3** and posterior reductive elimination. 

## 3. Asymmetric Preparation of Quaternary C-CF_3_ Stereocentres on *β*-keto Esters

Nowadays the importance of the trifluoromethyl group is well known and appreciated. In pharmaceutical research, molecules bearing the CF_3_ group are of high interest because it generally encourages remarkable and unusual changes in the properties of the original drug (better metabolic stability, enhanced biodisponibility and modulated lipophilicity) [1]. Since Lehmann reported in 1928 the first biologically active trifluoromethylated molecule [62], the trifluoromethyl group has gained more and more prominence. 

α-Trifluoromethyl *β*-keto esters are intriguing target molecules due to the presence of a chiral quaternary centre and the derivatizable carbonyl group. Thus, its enantiopure asymmetric preparation has been a thrilling challenge for the scientific community.

**Scheme 4:**
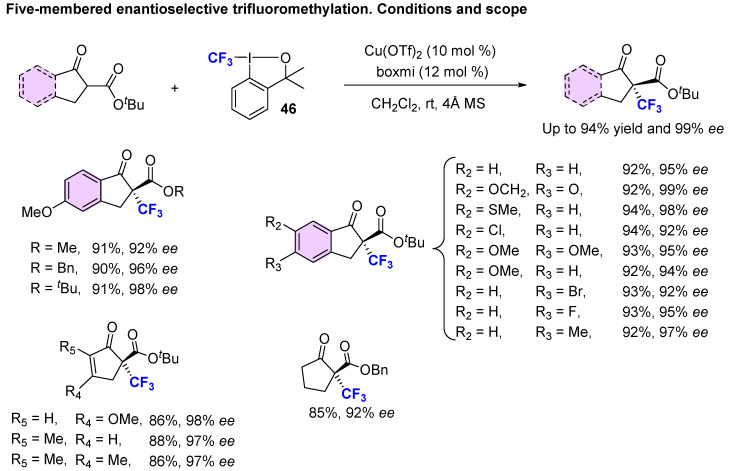

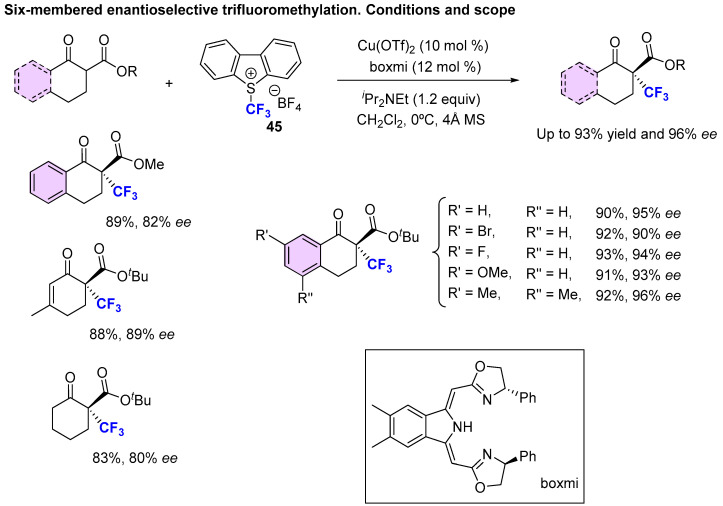
Reaction conditions and scope for the enantioselective electrophilic trifluoromethylation of five- and six-membered cyclic *β*-keto esters.

In the 80s [63] the first electrophilic trifluoromethylating reagents developed by Yagupol’skii appeared and later on in the 90s [64], Umemoto presented the second generation based on (trifluoromethyl)dibenzoheterocycle salts, being the *S*-(trifluoromethyl)dibenzthiophenium tetrafluoroborate, **44**, (Umemoto’s reagent, Scheme 4) the best known, but it was not until 2003 that Cahard and co-workers [65] applied these reagents in the preparation of non-asymmetric *α*-trifluoro-methyl *β*-keto esters.

### 3.1. Metal-Catalyzed Methods

Gade and co-workers [38] described in 2012 the enantioselective copper-catalysed trifluoromethylation of *β*-keto esters using commercially available trifluoromethylating reagents. The trifluoromethylation of 1-indanone derivatives proceeded best under Cu(OTf)_2_ catalysis in presence of bisoxazoline boxmi as a stereo-directing ligand and with the Togni hypervalent iodine reagent **46** (Scheme 4). The method tolerated a great range alkyl 1-indanones-2-carboxylates, either with electron-withdrawing or electron-donating groups in aromatic position, providing excellent enantioselectivities and yields. Primary esters also yielded the corresponding trifluoromethylated products with excellent enantioselectivities (up to 96% *ee*). The method was applied with success to *t*-butyl 2-oxocyclopentene-1-carboxylates and benzyl 2-oxocyclopentane-1-carboxylates. Despite the fact Togni’s reagent **46** was the best choice in the five-membered ring substrates, in order to achieve high enantiocontrol in the more enolizable six-membered ring *β*-keto esters, Umemoto’s reagent **45** was selected in presence of the non-nucleophilic base *^i^*Pr_2_NEt (Scheme 4). Unfortunately, acyclic *β*-keto esters were found to be unreactive under these reaction conditions.

**Scheme 5:**
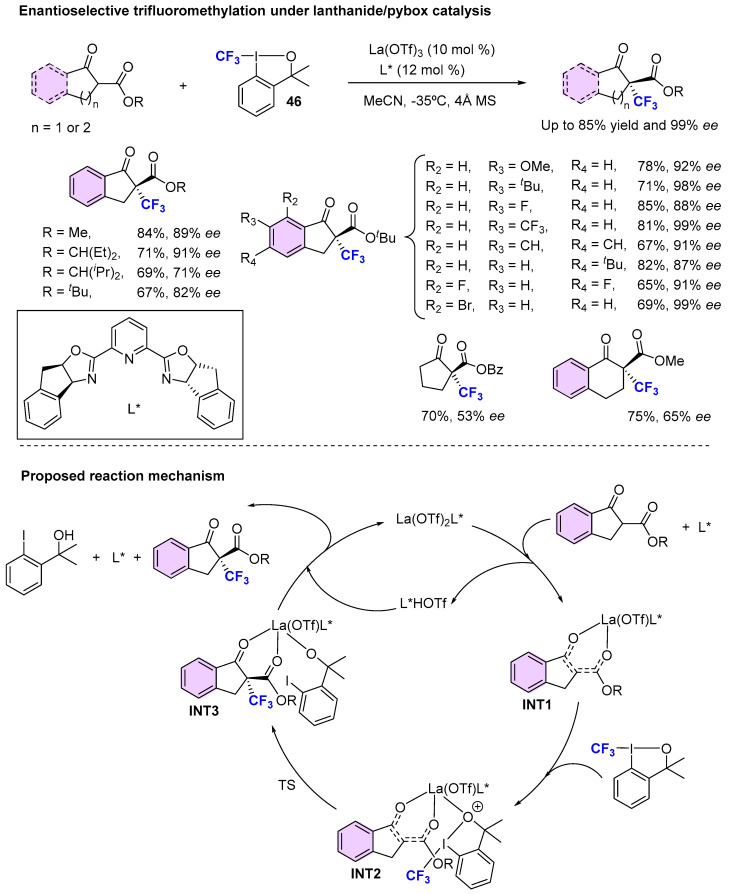
Electrophilic enantioselective *α*-trifluoromethylation of cyclic *β*-keto esters under La(III)/(*R,S*)-ind-pybox catalysis. Scope and proposed mechanism based on experimental and computational techniques.

Later, in 2019 Cossío and Vallribera [66] reported a highly enantioselective catalytic method for the synthesis of quaternary α-trifluoromethyl derivatives cyclic *β*-keto esters under lanthanide catalysis. The methodology implemented by these authors required the use of the Togni reagent **46** and the combination of La(OTf)_3_ and Nishiyama-type indanyl-pybox ligand, which are commercially available reagents. With the optimized pre-catalyst combination and reaction conditions in hand, a broad range of *β*-keto esters were examined. Best results were obtained with methyl 1-indanone-2-carboxylates, which was remarkable since it showed that this method could be applied to simple *β*-keto esters. In the presence of either electron-donating or electron-withdrawing groups excellent enantioselectivities were obtained. Finally, the more enolizable six-membered rings substrates such as alkyl 1-oxo-1,2,3,4-tetrahydronaphtalene-2-carboxylates and alkyl 2-oxocyclopentanone carboxylates gave lower *ee*. The mechanism was explored both by experimental and computational techniques. ESI-MS experiments and NMR studies determined the formation of **INT1** (Scheme 5) and the coordination of Togni’s reagent to the lanthanum. Reactions in presence of scavengers determined that the presence of trifluoromethyl radicals in the alkylation process could not be discarded. Afterwards, computational studies at the B3LYP-D3/6-31G*&LanL2DZ level of theory were carried out, and the coordination pattern of the cationic intermediate **INT2** in Scheme 5 revealed an efficient blockage of the prochiral *Si* face of the La^III^ enolate. The hindrance of the *Si* face of the substrate resulted in an efficient S_N_2-like saddle point TS, which consisted of a *Re* attack of the C_α_ atom of the enolate moiety on the CF_3_ group of the Togni reagent, forming **INT3**. Unfortunately, this catalytic method could not be applied to open-chain *β*-keto esters successfully.

### 3.2. Organocatalytic Methods

**Scheme 6:**
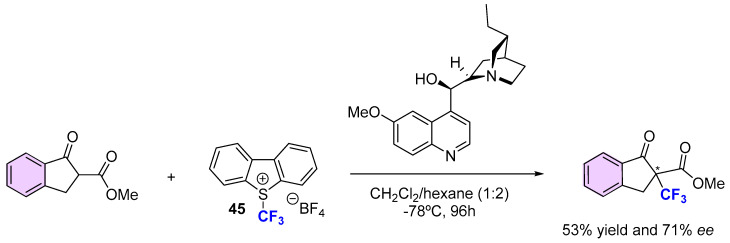
Asymmetric trifluoromethylation of methyl 1-indanone-2-carboxylate using the Umemoto’s reagent and hydroquinine.

In 2007, Cahard’s group reported the very first asymmetric preparation of α-trifluoromethylated cyclic *β*-keto esters under an organocatalyzed based methodology (Scheme 6) which required the use of *S*-(trifluoromethyl)dibenzthiophenium tetrafluoroborate **45** as electrophilic reagent and a cinchona alkaloid based chiral phase-transfer catalysts (PTC) [67]. They noticed that using a quaternary ammonium PTC and potassium carbonate, the *ee* were not higher than 20%. The formation of potassium *β*-keto ester enolate was responsible of the low *ee*. Thus, to banish the presence of achiral enolates the use of the tertiary PTC hydroquinine was the solution giving the expected α-trifluoromethylated *β*-keto ester in 53% yield with 71% *ee*. 

**Scheme 7:**
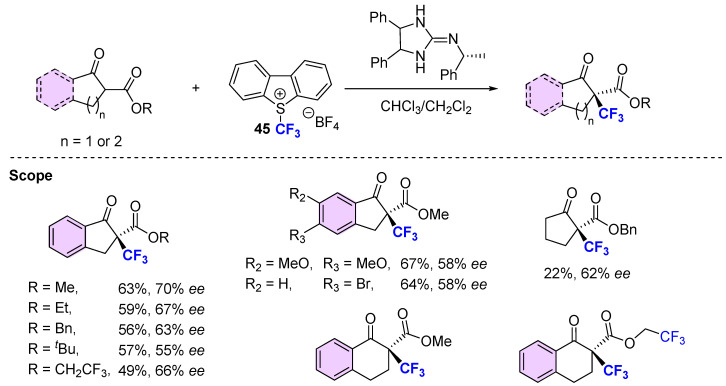

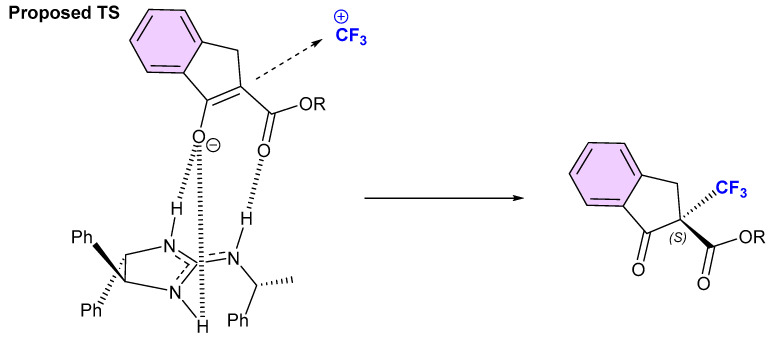
Asymmetric organocatalytic trifluoromethylation developed by Shibata and the proposed transition state (TS).

A few years later, Shibata [68] deeply investigated the use of chiral guanidines with *S*-(trifluoromethyl)dibenzothiophenium tetrafluoroborate **45**. These guanidines act as Brønsted bases to generate chiral guanidinium enolates of *β*-keto esters, which attack the electrophilic trifluoromethylating reagent in a stereospecific manner. The optimization of the reaction included solvents, temperature, electrophilic CF_3_ source and six different guanidines (Scheme 7). Mixtures of chlorinated solvents were the best option to achieve high enantioselectivities and yields at very low temperature. The electrophilic power of Umemoto’s reagents strongly influenced the reaction. Thus, the selenophenium analogous was not appropriate and even the dinitro derivative of Umemoto’s reagent gave lower results. Finally, the most sterically encumbered guanidines did not provide higher *ee*, and the suppression of one H-bonding from the imidazolidine ring system dramatically affects the *ee*, yielding a racemic compound. Thus, this supports the transition state structure where the guanidinium enolates of *β*-keto esters coordinates the substrate through H-bonding for an excellent stereochemical CF_3_ transfer (Scheme 7). 

The scope of the reaction was investigated. The use of bulkier 1-indanone derivatives did not secure better enantioselectivities; the primary methyl ester provided the best results (63% yield and 70% *ee*). The presence of substituent’s in the aromatic ring with different electronic natures were also tolerated. The 1-tetralone series and benzyl cyclopentanone-2-carboxylate were in the same range of *ee* values but lower yield (Scheme 7).

**Scheme 8:**
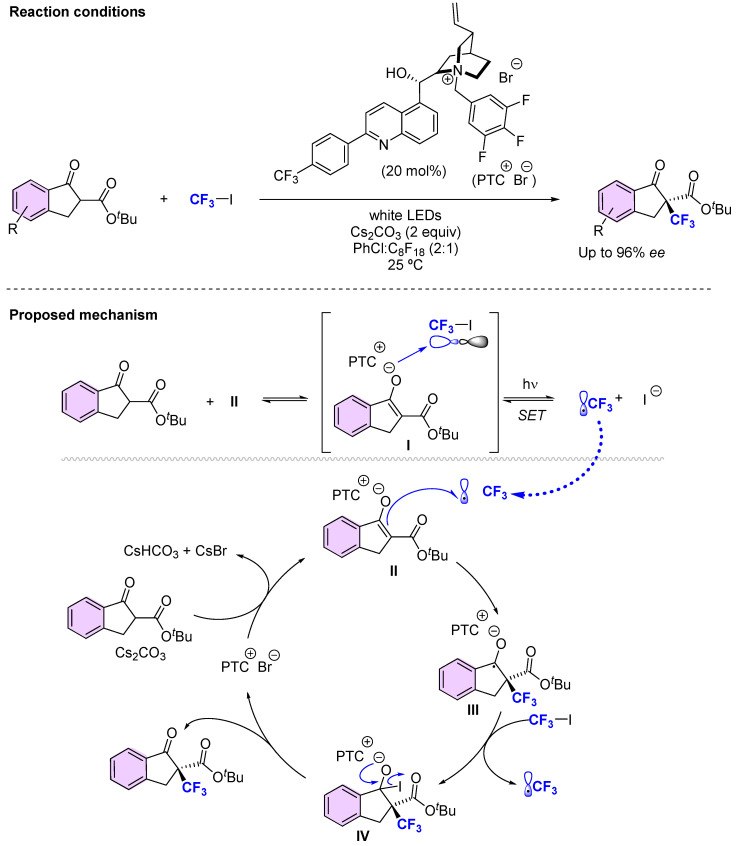
Conditions and mechanism for the visible-light-driven PTC catalyzed enantioselective perfluoroalkylation and trifluoromethylation of cyclic *β*-keto esters assembled by Melchiorre.

In 2015, Melchiorre’s research group [69] developed an interesting enantioselective perfluoroalkylation and trifluoromethylation of alkyl 1-indanone-2-carboxylates (Scheme 8) combining both visible-light and phase-transfer-catalysts (PTC). The methodology was based on the in situ formation of the photochemical active electron donor–acceptor (EDA) complexes from chiral ammonium enolates and perfluoroalkyl iodides. The catalyst optimization revealed that stereocontrol was sensitive to structural modifications at the 2′ position of the quinoline ring, being the organocatalyst PTC^+^Br^−^ (Scheme 8) the one which provided the best results in terms of *ee*. The synthetic application of this method was tested, assaying different substituted indanone-derived *β*-keto esters with methyl iodide. Notably, trifluoromethyl-containing quaternary stereocentres could be easily prepared reacting *β*-keto esters with methyl iodide under the same conditions, achieving *ee* up to 96%.

Based on some diagnostic mechanistic experiments, a radical chain propagation pathway was proposed. The trifluorometil radical could be generated upon irradiation of the chiral EDA complex. Then, the electrophilic CF_3_**^•^** reacted with the chiral enolate of the *β*-keto ester (**II** in the scheme below) giving place to the C-C bond formation through a stereo-controlled manner. The resulting species (**III**, in Scheme 8, vide infra) abstracted an iodine atom form the perfluorinated iodine compound, giving a new CF_3_**^•^** and the α-trifluoromethyl *β*-keto ester final product after the elimination of the iodide anion. Years later, Li and collaborators [70] found that EDA complex were responsible for the visible light absorption and the PTC catalyst interacted by electrostatic interactions with the *β*-keto ester enolate by DFT calculations.

## 4. Asymmetric Preparation of Quaternary C-SCF_3_ Stereocentres on *β*-keto Esters

In recent years, considerable attention has been focused on the trifluoromethylthio group (-SCF_3_) due to its unique properties of remarkable electron-withdrawing behaviour, excellent metabolic stability and high lipophilicity [71]. In fact, all these properties allow the trifluoromethylthiolated therapeutic molecules across lipidic membranes easily, which is crucial for the biodisponibility of the drug. Because of the high interest in SCF_3_ containing molecules, well-built methods for their insertion into organic molecules have been widely investigated. Specifically, both the racemic and the enantioselective trifluoromethylthiolation reaction on *β*-keto esters have been widely explored. 

### 4.1. Metal-Catalyzed Methods

**Scheme 9:**
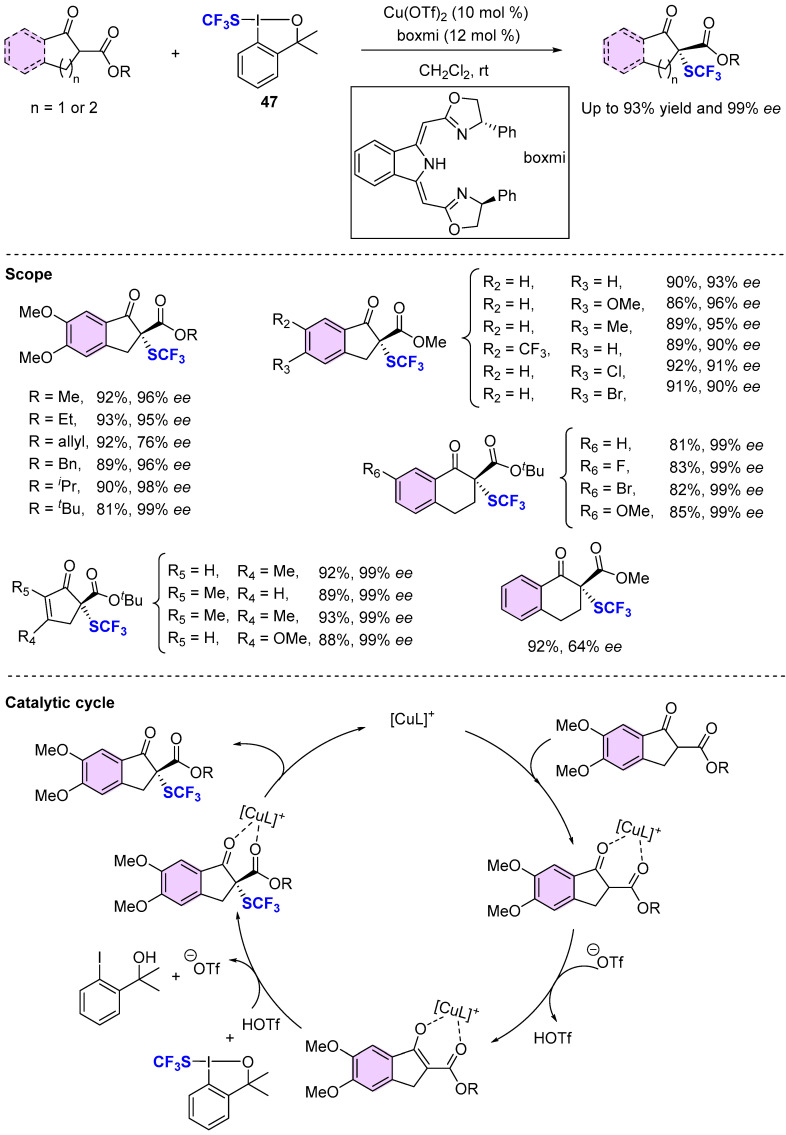
Copper-boxmi electrophilic trifluoromethylthiolation of several *β*-keto esters and the proposed catalytic cycle.

The work of Gade’s group [72] is the only example found in the literature, as far as we know, regarding the metal-catalyzed trifluoromethylthiolation of *β*-keto esters. The methodology consists on the use of chiral copper–boxmi complex (Scheme 9). They applied their broad experience in the use of this chiral pincer ligand in other reactions such as the asymmetric Fe-boxmi catalysed azidation of *β*-keto esters [73] and the asymmetric Cu-boxmi catalysed trifluoromethylation of *β*-keto esters [38] among others. With their optimized reaction conditions, consisting in using one equivalent of Lu and Shen’s hypervalent iodine reagent **47** [74] to transfer a –SCF_3_ group. A wide range of substrates possessing indanone, tetralone, cyclopentenone and cyclohexenone (one example, 99% *ee*) cores were well tolerated, obtaining in all cases excellent enantiomeric excess values (Scheme 9). In general, the size of the ester group it is not crucial for achieving high enantioselectivity. Several mechanistic experiments, including EPR spectroscopy and ^19^F-NMR led the authors to propose a catalytic cycle (Scheme 9). The combination of copper (II) with boxmi ligand coordinates to the *β*-keto ester substrate, which is deprotonated by a triflate anion. 

The triflic acid in situ acts as an activator of the hypervalent trifluoromethylthiolated iodine reagent **24**, generated which is attacked by the *β*-keto ester enolate through the more accessible *Re* face (the *Si* face is efficiently blocked by boxmi). The product is evacuated of the catalytic cycle generating again the chiral catalyst (Scheme 9).

### 4.2. Organocatalytic Methods

**Scheme 10:**
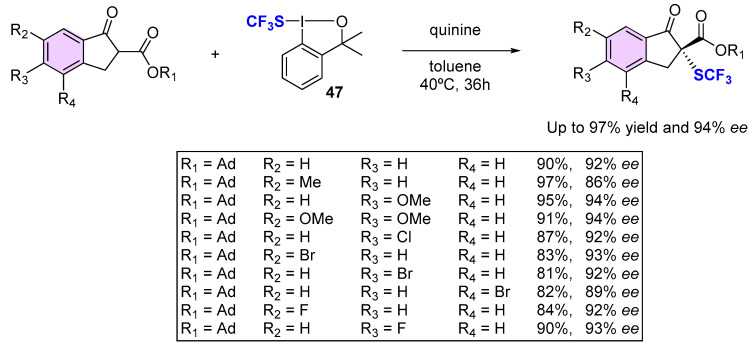
Organocatalyzed trifluoromethylthiolation of adamantyl 1-indanone-2-carboxylates. Reaction conditions and scope.

The first organocatalyzed enantioselective methodology reported for the introduction of -SCF_3_ onto the *α*-position of a *β*-keto esters was developed independently in 2013 by Shen and Rueping. Shen’s group [75] reported the use of trifluoromethylthiolated hypervalent iodine **47** and a catalytic amount of quinine in toluene at 40 °C in the reaction of a series of adamantyl 1-indanone-2-carboxylates (Scheme 10). The reactions of substrates with electron-withdrawing groups at the aromatic ring occurred with similar enantioselectivity to those of substrates with electron-donating groups, whereas the size of the ester group had a high impact on the enantioselectivity (Scheme 10). Interestingly, the methodology could be successfully applied to the adamantyl cyclopentanone *β*-keto ester (95% yield, 94% *ee*), but was not useful with larger ring size, such as tetralone derivatives.

**Scheme 11:**
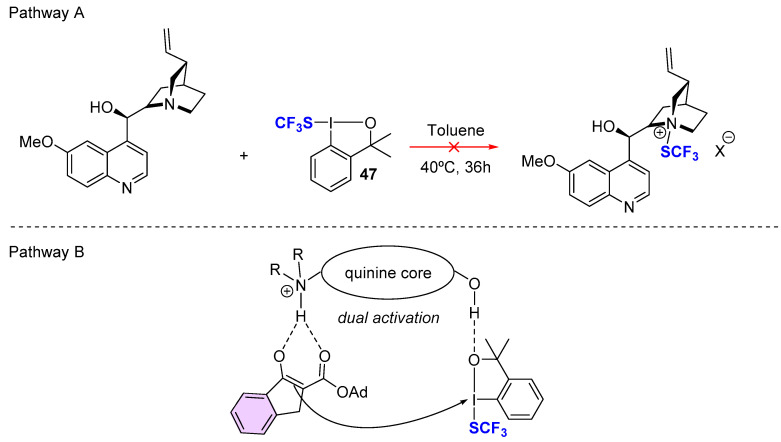
Proposed mechanisms for the quinine-catalyzed asymmetric trifluoromethylthiolation reaction.

In order to understand the differences in terms of enantioselectivity on these substrates, Shen proposed that the mechanism could proceed through two different pathways (Scheme 11). The first pathway (A) is based on the transfer of the SCF_3_ group from the hypervalent iodine reagent to the quinine, allowing the formation of a new SCF_3_-substituted quinine electrophile, which would be attacked by the nucleophilic enolized *β*-keto ester. The stochiometric mixture of Shen’s reagent **47** and quinine in toluene monitored by ^19^F-NMR ruled out this pathway, because the formation of new SCF_3_-substituted quinine electrophile was not evidenced. This experimental feature made the pathway B gain prominence, which consisted on a dual activation through simultaneous hydrogen bonding of the substrate and **47** to quinine (Scheme 11). This mechanism is steady since other cinchona alkaloids without the free OH group showed low reactivity. Moreover, this proposal is sterically congested and justifies the lack of reactivity of tetralone and 1-benzosuberone-derived *β*-keto esters. These more enolizable substrates form nonplanar enolates that are not able to generate the dual-activated intermediate, while the 1-indanone enolates are planar and fit very well in this model.

**Scheme 12:**
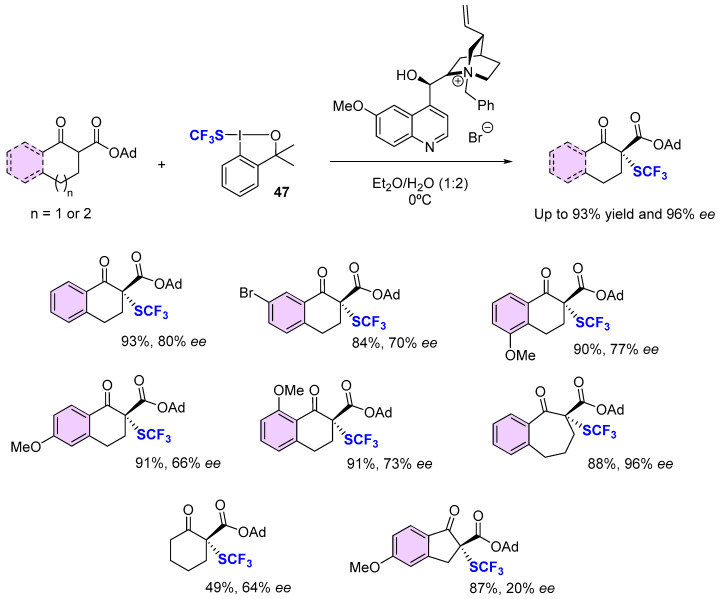
Scope of asymmetric trifluoromethylthiolation of *β*-keto esters mediated by PTCs.

Then, to expand the scope to the more enolizable 1-tetralone or 1-benzosuberone-derived *β*-keto esters, they assayed cinchona alkaloids based chiral phase-transfer catalysts, yielding the trifluoromethylthiolated quaternary *β*-keto esters in great yields and enantioselectivities (Scheme 12). Interestingly enough, the reaction of the indanone derivatives through the use of PTC occurred with low enantioselectivity, in contrast to the high enantioselectivity obtained when using quinine as catalyst. Despite the huge efforts done by Shen’s group, the methodology could not be applied to acyclic *β*-keto esters.

**Scheme 13:**
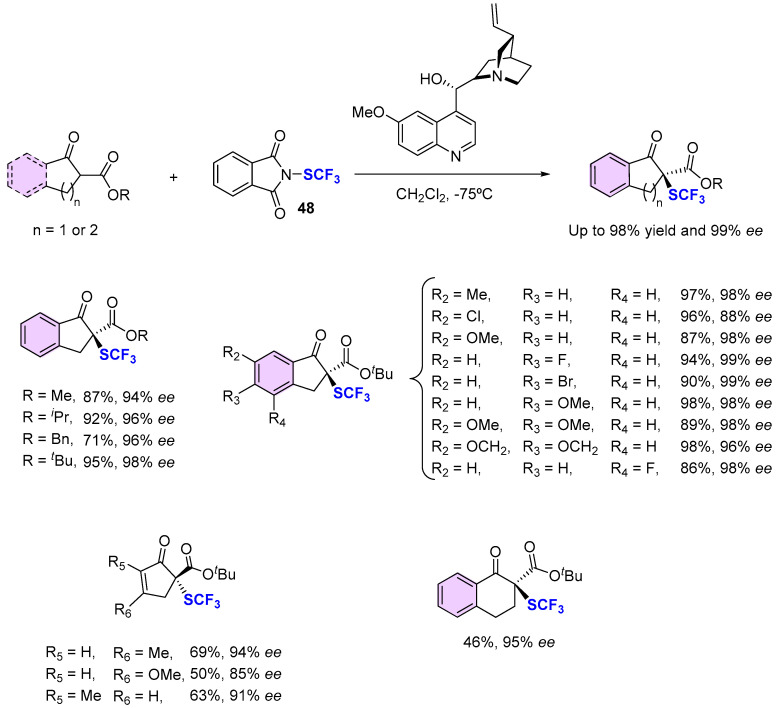
Scope of the enantioselective trifluoromethylthiolation.

At the same time as this previous work, Rueping’s group [76] reported an organocatalytic scenario using quinidine as catalyst in dichloromethane and *N*-trifluoromethylthiophtalimide **48** as electrophilic -SCF_3_ source, an air and moisture stable reagent. The reaction gave excellent yields and enantioselectivities (Scheme 13). In general, the reactions of cyclic 1-indanone derivatives bearing various electron-donating and electron-withdrawing substituents in different positions of the aromatic ring proceeded successfully to provide the corresponding products in high yields and excellent enantioselectivities (88–99% *ee*; Scheme 13). Furthermore, cyclopentenone *β*-keto esters underwent trifluoromethylthiolation under these conditions in moderate yields but excellent enantioselectivites. Additionally, this methodology accepted 1-tetralone based *β*-keto ester in a 46% yield and 95% *ee*. 

**Scheme 14:**
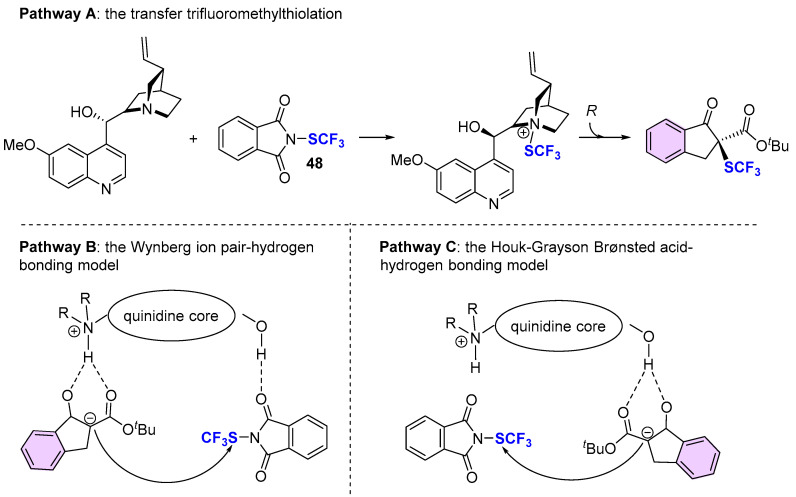
Possible models for the Rueping’s asymmetric electrophilic trifluoromethylthiolation.

Four years later, in 2017 Xue [77] wanted to cast some light into the mechanism of Rueping’s asymmetric electrophilic trifluoromethylthiolation reaction using density functional theory studies. The Houk-Grayon is the commonly preferred model [78] for cinchona alkaloid-catalysed reactions, however after the evaluation of three different mechanistic scenarios (pathways A, B and C, Scheme 14), the calculations showed that the most preferred model was the Wynberg ion pair-hydrogen bonding model [79] (pathway B in Scheme 14) in which the SCF_3_ transfer from *N*-trifluoromethylthiophtalimide **25** to *β*-keto ester proceeds via an S_N_2-like saddle point transition state.

**Scheme 15:**
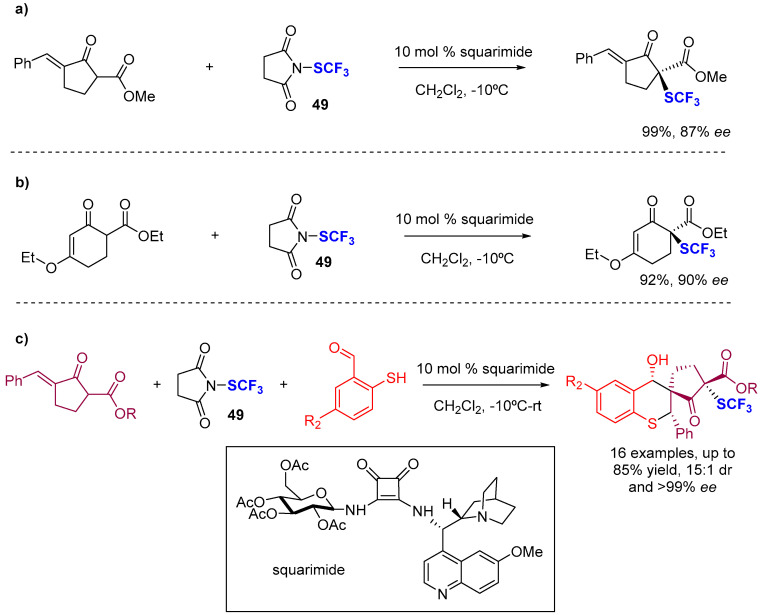
(**a**,**b**) Enantioselective trifluoromethylthiolation of cycloalkenones driven by squaramide catalyst. (**c**) Bifunctional squaramide-catalyzed electrophilic trifluoromethyl-thiolation−sulfur−Michael/aldol cascade reaction for the construction of -SCF_3_ containing spiro-cyclopentanone−thiochromanes.

Du and collaborators, [80] reported in 2017 the asymmetric electrophilic trifluoro-methylthiolation of (*E*)-alkyl 3-benzylidene-2-oxocyclopentane carboxylates cyclopentanone using a bifunctional squaramide as organocatalyst and *N*-trifluoromethylthiosuccinimide (**49**) as an electrophilic reagent (Scheme 15a). After some experimentation, the squaramide derived from hydroquinine and D-glucopyranose *(*Scheme 15) afforded the most efficient catalyst. The same conditions were extended to ethyl 4-ethoxy-2-oxocyclohex-3-ene-1-carboxylate in high yield and excellent *ee* (Scheme 15b). Additionally, the reaction of (*E*)-alkyl 3-benzylidene-2-oxocyclopentane carboxylates with **49** in presence of 2-mercapto-5-substituted benzaldehydes allowed the formation of a series of chiral *spiro*-cyclopentanone thiochromanes bearing an SCF_3_ group through a one pot electrophilic trifluoromethylthiolation−sulfur−Michael/aldol cascade reaction (16 examples, 92–99% *ee*, Scheme 15c). 

### 4.3. Methods Involving Chiral -SCF_3_ Reagents

**Scheme 16:**
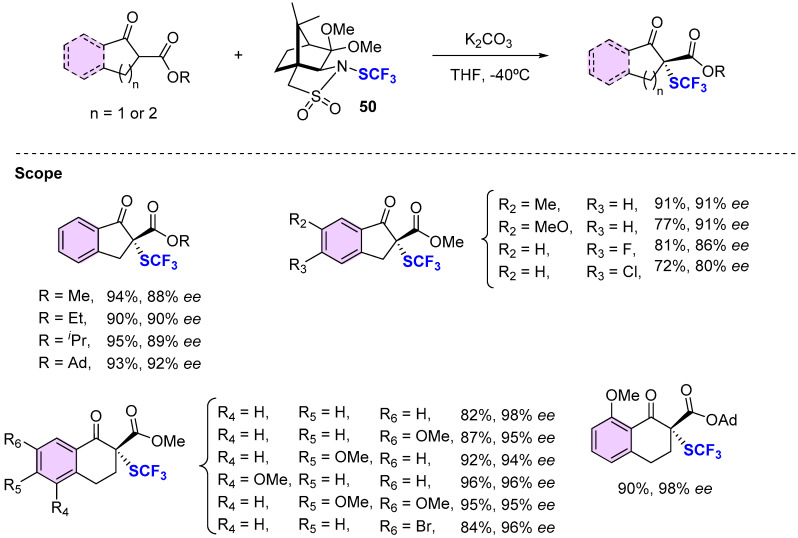
Asymmetric trifluoromethylthiolation of 1-indanone and 1-tetralone *β*-keto esters using the chiral SCF_3_ reagent **50**.

The benefits of transferring a group from a chiral reagent to a prochiral substrate to give the enantiomerically pure product are indubitable, mainly because it offers a simple and straightforward strategy. For this reason, Shen and co-workers [81] put their efforts on designing new optically pure electrophilic -SCF_3_ containing reagents that could allow reliable methods for the synthesis of enantiomerically pure organic compounds. This field complements all the previous trifluoromethylthiolation strategies explained up to this point. A new family of optically pure (1*S*)-(−)-*N*-trifluoromethylthio-2,10-camphorsultam, and derivatives were synthesized and tested. The dimethoxycamphorsultam derivative **50** in THF, at −40 °C and in the presence of K_2_CO_3_ proved excellent for the asymmetric introduction of SCF_3_ into 1-indanone and 1-tetralone *β*-keto esters (Scheme 16). These conditions tolerated the presence of electron-donating groups in the aromatic ring, nevertheless for achieving high *ee* in the cases of less nucleophilic substrates (presence of electron-withdrawing groups in the aromatic ring) the reaction requires a higher temperature. Moreover, the effect of the steric hindrance of the ester group was significant for achieving high *ee* values.

## 5. Conclusions

During the past decade, both metal-catalyzed and organocatalyzed asymmetric processes have been devised, making asymmetric fluorination, trifluoromethylayion and trifluorothiomethylation a useful tool. Asymmetric fluorination of *β*-keto esters has been successfully achieved by different research groups in alkyl 1-indanone 2-carboxylates and alkyl 1-oxo-1,2,3,4-tetrahydronaphtalene-2-carboxylate substrates, independently of the bulkiness of the ester group. In the case of alkyl 2-oxocyclopentane-1-carboxylates and the corresponding cyclohexanes fluorinated products are generally obtained in lower *ee*. Moreover, acyclic *β*-keto esters are the most difficult *β*-keto esters substrates in this reaction in terms of reactivity. Notably, Ti/TADDOL complexes of Togni’s group was useful for a larger number of acyclic compounds. Several different excellent organocatalysts systems have been developed for 1-indanone derivatives; however new organocatalytic systems are still needed for 1-tetralone and acyclic *β*-keto esters derivatives. Generally NFSI is the selected reagent for asymmetric fluorinations.

In the case of nucleophilic reagents, remarkable results have been obtained for 1-indanone derivatives. However, new approaches that permit increase more the scope of substrates are still needed. Organocatalyzed fluorination of *β*-keto esters has proved to be highly efficient, bifunctional chiral thioureas derived from cinchona alkaloids giving really interesting results. 

Different successful asymmetric metal-catalyzed trifluoromethylation approaches have been described by Gade’s and Vallribera’s groups. The results are excellent for 1-indanone derivatives. However, poor results have been obtained in the enantioselective fluorination of alkyl 2-oxo-cyclopentane and 2-oxocyclohexane-1-carboxylates. New metal-catalyzed methods also need to be developed for acyclic *β*-keto esters. In the trifluoromethylation cases, metal-catalyzed are superior to organocatalyzed described methods. However, combining both visible-light and phase-transfer-catalysts seems a good choice. Moreover, the design of new efficient chiral trifluoromethylating reagents is still needed.

Remarkable results have been obtained by Gade’s group in the asymmetric metal-catalyzed trifluoromethylthiolation. Competitive results were achieved with cinchone and squarimide derivatives as organocatalyts. Notably, the design and use of (1*S*)-(−)-*N*-trifluoromethylthio-2,10-camphorsultam chiral reagent in enantioselective trifluoromethylthiolation reactions resulted an excellent option.

Generally, in terms of reagents, organocatalytic methods are more affordable than metal catalyzed methods mainly due to the cost of the chiral ligands. Finally, the creation of new fluorinated compounds is expected to remain a major focus of interest next years and therefore still more new fluorinating, trifluoromethylating and trifluoromethylthiolating processes will be required.
